# The effects of irradiation dose and storage time following treatment on the viscoelastic properties of decellularised porcine super flexor tendon

**DOI:** 10.1016/j.jbiomech.2017.04.005

**Published:** 2017-05-24

**Authors:** Anthony Herbert, Jennifer H. Edwards, Gemma L. Jones, Eileen Ingham, John Fisher

**Affiliations:** a (IMBE) Institute of Medical and Biological Engineering, School of Mechanical Engineering, University of Leeds, Leeds, UK; b IMBE, Faculty of Biomedical Sciences, University of Leeds, Leeds, UK

**Keywords:** Tendon, Decellularised, Anterior cruciate ligament, Tissue engineering, Irradiation

## Abstract

Decellularised porcine super flexor tendon (pSFT) offers a promising solution to the replacement of damaged anterior cruciate ligament. It is desirable to package and terminally sterilise the acellular grafts to eliminate any possible harmful pathogens. However, irradiation techniques can damage the collagen structure and consequently reduce the mechanical properties. The aims of this study were to investigate the effects of irradiation sterilisation of varying dosages on the viscoelastic properties of the decellularised pSFT.

Decellularised pSFT tendons were subjected to irradiation sterilisation using either 30 kGy gamma, 55 kGy gamma, 34 kGy E-beam, 15 kGy gamma, 15 kGy E-beam and (15 + 15) kGy E-beam (fractionated dose). Specimens then underwent stress relaxation testing at 0 and 12 months post sterilisation to determine whether any effect on the viscoelastic properties was progressive.

Significant differences were found which demonstrated that all irradiation treatments had an effect on the time-independent and time-dependent viscoelastic properties of irradiated tendons compared to peracetic acid only treated controls. No significant differences were found between the irradiated groups and no significant differences were found between groups at 0 and 12 months. These results indicate the decellularised pSFT graft has a stable shelf-life.

## 1 Introduction

The anterior cruciate ligament (ACL) is considered to be the primary, passive stabiliser of the knee (Kiapour and Murray, 2014), contributing to restricting anterior displacement of the femur relative to the tibia (Samuelsson et al., 2009). Once ruptured, a full replacement of the structure is required to restore stability and function to the knee. Traditionally, this involves the use of autologous or allogeneic graft tissue, however these treatment options have limitations such as donor site morbidity and lack of availability respectively (Herbert et al., 2016). Our group has recently developed a decellularised porcine super flexor tendon (pSFT) graft, a biological scaffold which offers the benefit of availability “off the shelf” (Jones et al., 2016). During the development of the pSFT graft, peracetic acid was used to achieve sterilisation since this chemical sterilant is effective in eliminating resistant bacterial spores in decellularised biological scaffolds (Wilshaw et al., 2006). However, the use of chemical sterilisation is not ideal because the product would require aseptic packaging post-sterilisation with the risk of contamination. Ideally, the use of a terminal sterilisation process, such as irradiation is required.

Sterilisation using irradiation, however, has inherent drawbacks, particularly when applied to biological tissues. This includes free-radical mediated damage to the collagenous structure, leading to a reduction in the mechanical properties (Smith and Kearney, 1996). This has been reported to occur in human tissues after standard dose 25 kGy gamma irradiation (Smith and Kearney, 1996, Smith et al., 1996, Ren et al., 2012), although it has been reported that fractionated doses of irradiation do not affect structural and biomechanical properties (Hoburg et al., 2011, Wei et al., 2013). In an initial study of the effects of a range of doses and irradiation types (30 kGy gamma, 55 kGy gamma, 34 kGy E-beam, 15 kGy gamma, 15 kGy E-beam and 15 + 15 kGy E-beam [fractionated dose]) on the decellularised pSFT, we demonstrated small dose related increases in denatured collagen levels, reduced thermal denaturation temperature, coinciding with reduced ultimate tensile strength and Young’s modulus (Edwards et al., 2016).

The viscoelastic properties of the tissues were however not explored in the previous study and these remain a key indicator of the functional performance of the grafts due to the long term repetitive loading that they would encounter when implanted in the knee. Hence, this study examined the effects of variation in irradiation treatment (as above) on the stress relaxation response of the decellularised pSFT. It was hypothesised that non dose-dependent results would also be observed for viscoelastic parameters derived from the stress relaxation data. Furthermore, this was examined at two time points, directly following treatment and after 12 months, to ascertain the stability of the decellularised pSFT scaffold upon storage.

## 2 Materials and methods

### 2.1 Tissue sourcing

Female ∼70 kg, 4 month old, Large White pigs were obtained from an abattoir (J. Penny, Leeds, UK) within 24 h of slaughter. Once removed, all pSFT’s were stored at −80 °C with phosphate buffered saline (PBS) soaked filter paper prior to decellularisation.

### 2.2 Decellularisation

Decellularisation was achieved using a 0.1% w/v SDS (sodium dodecyl sulphate) process refined to incorporate bioprocesses including bioburden reduction, fat reduction and terminal chemical sterilisation using 0.1% w/v peracetic acid (Jones et al., 2016), with least disruption to the biomechanical properties (Herbert et al., 2015). Following successful completion of the decellularisation process, tendons were transferred aseptically to foil packaging and stored at −80 °C.

### 2.3 Sterilisation

For pSFTs undergoing irradiation sterilisation, the decellularised pSFTs were removed from storage at −80 °C and shipped under refrigerated conditions to Synergy Health PLC (Swindon, UK). Six groups of packaged tendons (*n* = 12) were irradiated at a tolerance of ±10% using either 30 kGy gamma, 55 kGy gamma, 34 kGy E-beam, 15 kGy gamma, 15 kGy E-beam and 15 + 15 kGy E-beam (fractionated dose – 15 kGy E-beam applied twice). Each irradiation group was then subdivided into tendons for immediate analysis after irradiation treatment (t = 0 months; *n* = 6) and tendons for analysis after 12 months storage at 4 °C (t = 12 months; *n* = 6). Control decellularised pSFTs (PAA sterilised only) were also removed from −80 °C storage at time zero and analysed immediately (t = 0) or stored at 4 °C prior to analysis at 12 months (*n* = 6 in both cases).

### 2.4 Biomechanical testing

#### 2.4.1 Specimen preparation

For each group investigated, pSFT’s were removed from their packaging and immersed in dry ice to aid processing them into dumbbell shapes with a working cross-sectional area of approximately 3.5 × 5 mm and gauge length of 30 mm. The width and length of each specimen was measured at three points using a Vernier callipers and averages calculated. An average value for the thickness was similarly calculated using a thickness gauge under a constant force of 0.65 N. All specimens were then wrapped in PBS soaked filter paper and allowed to thaw and equilibrate at room temperature for at least two hours prior to mechanical testing.

#### 2.4.2 Stress relaxation testing

Stress relaxation testing was carried out using a method previously employed by our group (Herbert et al., 2015). In brief, specimens were mounted via bespoke cryogrips to an Instron 3365 (Instron, Bucks, UK) materials testing machine equipped with a 500 N load cell. Specimens were then tensioned to a pre-load of 0.5 N, followed by 10× preconditioning cycles between 0 and 5% strain at a rate of 15 mm/min. A ramp and hold cycle was then applied consisting of a ramp phase at a rate of 30 mm/min until a stress of 5 MPa was achieved. The specimens were then held at the strain developed at the end of the ramp phase for a period of 300 s whilst stress relaxation occurred. Data was recorded at a frequency of 10 Hz.

Engineering stress (*σ*) was calculated by the dividing the force recorded by the load cell by the working cross-sectional area of the specimen, whereas engineering strain (*ε*) was determined by dividing the crosshead displacement by the gauge length of the specimen.

The relaxation modulus (*E*(*t*)) was calculated using the following:

$$E(t)=\frac{\sigma (t)}{\varepsilon }$$

and fitted (r^2^ > 0.97) to a modified Maxwell-Wiechert model using the non-linear least squares method (Jimenez Rios et al., 2007);

$$E(t)=E_{0}+\frac{1}{t_{0}}\overset{n}{\underset{i=1}{\sum }}E_{i}\tau_{i}\cdot \exp^{\left(-\frac{t}{\tau_{1}}\right)}\left(\exp^{\left(\frac{t_{0}}{\tau_{i}}\right)}-1\right)$$

The modification accounts for any stress relaxation that may have occurred during the ramp phase (*0* *≤* *t* *≥* *t_0_*). The simplest form of the model consists of two Maxwell elements in parallel with a single spring (i.e. *n* = 2). *E_0_* is the time-independent elastic modulus of the single spring, whereas *E_i_* and *τ_i_* represent the time-dependent elastic modulus and relaxation time respectively of the Maxwell elements.

### 2.5 Statistical analyses

Statistical variances between groups were determined by two-way analysis of variance (ANOVA). Tukey’s honesty significant difference test was used for post hoc evaluation and a p-value of <0.05 was considered to be statistically significant.

## 3 Results

**Fig. 1:**
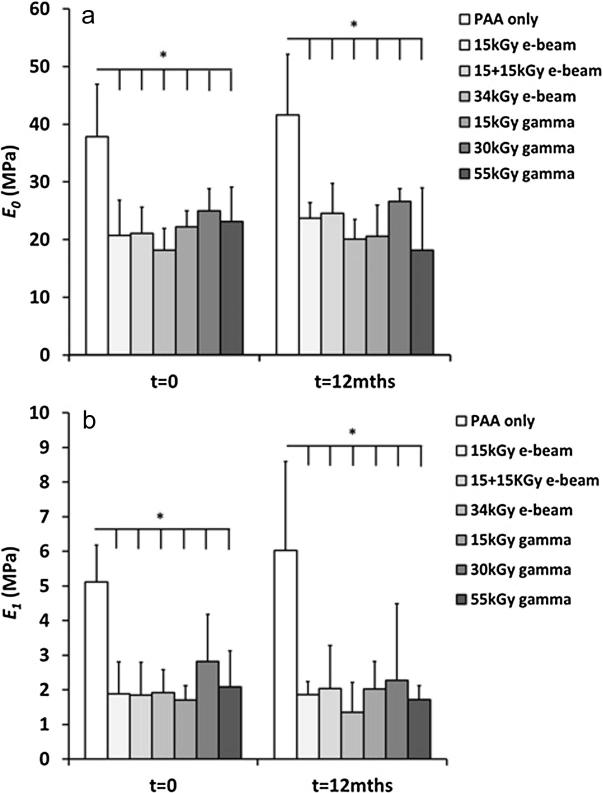
(a) The time-independent modulus, E_0_ and (b) the short term time-dependent modulus, E_1_ for all groups investigated at 0 & 12 months (mean ± 95% CI). *Indicates a significant difference (2-way ANOVA with Tukey post hoc analysis).

All irradiation treatments had a significant effect on the viscoelastic properties of the decellularised pSFT grafts compared to the chemically sterilised (PAA only treated) specimens. There was not only a significant reduction in the time-independent elasticity (*E_0_*), but also in the short term elastic response (*E_1_*) of all irradiated specimens (Fig. 1a and b respectively). Interestingly no significant differences were found between any of the irradiated groups, indicating that the reduction in these viscoelastic parameters was not a function of the irradiation type or dose investigated (Table 1). There were no significant differences found between any of the groups for the remaining parameters *E_2_* and *τ_1_* & *τ_2_*. Lastly, two way analysis of variance revealed that there was no significant interaction between the test groups with time and that there were no significant differences between groups at 0 and 12 months.

**Table 1:** Results from stress relaxation testing (mean ± 95% CI). *Indicates a value significantly different from PAA only control (2-way ANOVA with Tukey post hoc analysis).

Time	Group	E_0_ (MPa)	E_1_ (MPa)	E_2_ (MPa)	τ_1_ (MPa)	τ_2_ (MPa)
0 M	PAA only	37.80 ± 9.14	5.11 ± 1.07	3.88 ± 1.37	5.35 ± 0.74	115.72 ± 6.54
	15 kGy e-beam	20.71 ± 6.11 (*)	1.88 ± 0.92 (*)	2.37 ± 1.46	6.57 ± 1.48	99.50 ± 14.22
	15 + 15 kGy e-beam	21.08 ± 4.55 (*)	1.84 ± 0.95 (*)	1.68 ± 0.92	6.16 ± 1.12	133.42 ± 22.86
	34 kGy e-beam	18.18 ± 3.73 (*)	1.92 ± 0.66 (*)	2.73 ± 1.94	6.12 ± 1.31	128.46 ± 39.43
	15 kGy gamma	22.21 ± 2.75 (*)	1.71 ± 0.41 (*)	2.02 ± 0.59	5.43 ± 1.74	125.92 ± 14.93
	30 kGy gamma	25.00 ± 3.84 (*)	2.82 ± 1.36 (*)	3.16 ± 1.52	5.96 ± 1.00	115.41 ± 10.84
	55 kGy gamma	23.16 ± 5.91 (*)	2.08 ± 1.04 (*)	3.57 ± 1.37	5.42 ± 0.70	125.02 ± 10.17

12 M	PAA only	39.29 ± 11.53	5.72 ± 2.66	3.69 ± 2.18	5.03 ± 0.97	123.26 ± 20.82
	15 kGy e-beam	23.73 ± 2.64 (*)	1.86 ± 0.38 (*)	2.02 ± 0.69	5.93 ± 0.55	131.31 ± 20.14
	15 + 15 kGy e-beam	24.54 ± 5.21 (*)	2.04 ± 1.24 (*)	2.12 ± 1.05	5.21 ± 0.55	123.81 ± 30.20
	34 kGy e-beam	20.08 ± 3.41 (*)	1.34 ± 0.87 (*)	3.65 ± 2.08	5.20 ± 2.20	137.75 ± 17.80
	15 kGy gamma	20.57 ± 5.38 (*)	2.03 ± 0.78 (*)	3.68 ± 1.72	6.09 ± 0.94	128.11 ± 22.46
	30 kGy gamma	26.59 ± 2.23 (*)	2.27 ± 2.22 (*)	2.24 ± 1.76	5.17 ± 4.06	121.39 ± 80.60
	55 kGy gamma	18.19 ± 10.74 (*)	1.71 ± 0.40 (*)	2.62 ± 4.08	5.21 ± 1.51	119.10 ± 5.27

## 4 Discussion

Irradiation is a popular terminal sterilisation choice for medical devices. Doses of 25 kGy gamma are typically used for allografts, however 15 kGy can be used should there be a very low initial bioburden (ISO 11137-2-212). This would be the case for the decellularised pSFT following PAA treatment. This study aimed to determine the consequences of various irradiation doses and types (30 kGy gamma, 55 kGy gamma, 34 kGy E-beam, 15 kGy gamma, 15 kGy E-beam and 15 + 15 kGy E-beam [fractionated dose]) on the viscoelastic performance of the decellularised pSFT, a potential off-the-shelf ACL replacement graft.

Stress relaxation testing was performed and it was found that all irradiation groups had significantly reduced viscoelastic parameters when assessed using a modified Maxwell-Wiechert model (Jimenez Rios et al., 2007). More specifically, the time-independent elasticity (*E_0_*) and the short term time-dependent response (*E_1_*) were found to be significantly reduced following irradiation treatment in all cases at both 0 and 12 months. Interestingly, there were no differences between the irradiated groups, indicating that the reductions observed were dose independent. These findings were surprising as larger doses of irradiation doses might be expected to cause more damage to the tissue structure through mechanisms such as free radical mediated chain scission (Salehpour et al., 1995). Hence, any reduction in biomechanical performance might be expected to be similarly dose-dependent.

In our previous study, the standard material properties (e.g. tensile strength, Young’s modulus) of decellularised pSFT’s treated using the same types and doses of irradiation as reported here were determined. Reductions in these material properties were also demonstrated to be independent of irradiation type or dose (Edwards et al., 2016). It should be noted that the reduced material parameters were still more than sufficient for a graft to replace the native human ACL (Edwards et al., 2016). The denatured collagen content and thermal transition temperature of the pSFT were also examined and a clear dose-dependency was demonstrated, with greater amounts of denatured collagen and lower transition temperatures in the groups treated with higher doses of irradiation. It was postulated that the formation of new irradiation mediated crosslinks may have off-set the effect of any structural damage that occurred (Edwards et al., 2016). It is highly likely that the same mechanisms explain why there was no dependence of changes in the viscoelastic parameters upon irradiation type or dose.

Decellularisation has been shown to cause a reduction in both the time dependent and time independent moduli of the pSFT through providing an open matrix free of cells and ground substance for fluid flow and altering the collagen crimp pattern (Herbert et al., 2015). The data presented here indicates that irradiation sterilisation can reduce these parameters further, through further expansion of the ECM structure (affecting *E_0_*), allowing the ingress of more fluid and less viscous resistance (affecting *E_1_*). Although irradiation clearly affects the viscoelastic properties of the decellularised pSFT, it is important to note that the irradiation treated grafts retained the biomechanical properties necessary to function as a replacement ACL. This pertains to the viscoelastic properties examined in this study, but also the Young’s modulus and tensile strength (Edwards et al., 2016). In the short term, the changes to the matrix structure may favour endogenous cell migration and constructive remodelling of the decellularised pSFT.

Identifying a suitable terminal sterilisation method and demonstrating shelf-life are important factors in the translation of the decellularised pSFT to an off-the-shelf ACL replacement. It was hypothesised that there would be a non dose-dependent effect on the viscoelasticity and this hypothesis was proven. Irradiation at 25 kGy is recommended for the sterilisation of medical products by the IAEA (International Atomic Energy Agency) (Ren et al., 2012). Due to the dose independent effects on the viscoelasticity of the decellularised pSFT, this minimum dose appears to be appropriate. In addition, the viscoelasticity remained stable after 12 months storage at 4 °C, indicating that further degradation of the decellularised pSFT did not occur.

## Conflict of interest

J. Fisher and E. Ingham are consultants to and shareholders of Tissue Regenix Group plc. J. Fisher is also a consultant to DePuy Synthes, Invibio, and Simulation Solutions.
